# Relationships among Food Label Use, Motivation, and Dietary Quality

**DOI:** 10.3390/nu7021068

**Published:** 2015-02-05

**Authors:** Lisa M. Soederberg Miller, Diana L. Cassady, Elizabeth A. Applegate, Laurel A. Beckett, Machelle D. Wilson, Tanja N. Gibson, Kathleen Ellwood

**Affiliations:** 1 Department of Human Ecology, University of California, Davis One Shields Avenue, Davis, CA 95616, USA; E-Mail: tngibson@ucdavis.edu; 2 Department of Public Health Sciences, University of California, Davis, One Shields Avenue Davis, CA 95616, USA; E-Mails: dlcassady@phs.ucdavis.edu (D.L.C.); labeckett@phs.ucdavis.edu (L.A.B.); mdwilson@phs.ucdavis.edu (M.D.W.); 3 Nutrition Department, University of California, Davis One Shields Avenue, Davis, CA 95616, USA; E-Mail: eaapplegate@ucdavis.edu; 4 Port Republic, MD 20676, USA; E-Mail: kenutr@comcast.net

**Keywords:** food choice, nutrition labels, motivation, attention relationships among food label use, motivation, dietary quality

## Abstract

Nutrition information on packaged foods supplies information that aids consumers in meeting the recommendations put forth in the US Dietary Guidelines for Americans such as reducing intake of solid fats and added sugars. It is important to understand how food label use is related to dietary intake. However, prior work is based only on self-reported use of food labels, making it unclear if subjective assessments are biased toward motivational influences. We assessed food label use using both self-reported and objective measures, the stage of change, and dietary quality in a sample of 392 stratified by income. Self-reported food label use was assessed using a questionnaire. Objective use was assessed using a mock shopping task in which participants viewed food labels and decided which foods to purchase. Eye movements were monitored to assess attention to nutrition information on the food labels. Individuals paid attention to nutrition information when selecting foods to buy. Self-reported and objective measures of label use showed some overlap with each other (*r* = 0.29, *p* < 0.001), and both predicted dietary quality (*p* < 0.001 for both). The stage of change diminished the predictive power of subjective (*p* < 0.09), but not objective (*p* < 0.01), food label use. These data show both self-reported and objective measures of food label use are positively associated with dietary quality. However, self-reported measures appear to capture a greater motivational component of food label use than do more objective measures.

## 1. Introduction

The Nutrition Facts panel (NFP) provides information that consumers can utilize in making informed decisions about food choices that are in keeping with the most recent 2010 Dietary Guidelines for Americans (DGA). Key recommendations from DGA highlight the need to reduce intake of certain food ingredients such as solid fats (saturated and trans fats) and increase intake of others including fiber and micronutrients such as vitamin D; thus, selecting and eating more nutrient dense foods. The NFP and the more recently introduced front of package (FOP) symbols provide consumers with details that, in principle, can guide them to make informed choices to meet dietary recommendations, but evidence that individuals take advantage of this information is unclear. One approach to addressing the question of whether individuals take advantage of food labels is to ask individuals whether they use nutrition information on the food label when deciding what to buy. Using this approach, the majority of individuals appear to take advantage of food labels. Data from the 2005–2006 National Health and Nutrition Examination Survey (NHANES) show that 61.6% of participants report using the Nutrition Facts panel [1]. Another approach is to link these reports to individuals’ dietary quality [1,2,3,4,5,6,7,8,9,10,11,12,13,14,15]. Generally, this body of research has shown that individuals who use food labels also have healthier diets. For example, self-reported food label use was related to increased fiber and iron intake [6] and lower dietary fat intake [7,8,9] as well as overall dietary quality, as assessed by the Healthy Eating Index (HEI) [4,11].

A third approach is to investigate how well individuals are able to use nutrition labels to select healthier foods. Reviews of the literature generally show that individuals are able to use labels to perform basic tasks [16,17,18]. Recently, this question has been directed at a particular type of nutrition label called FOPs that appears on the front of food packages [19,20,21,22,23,24]. FOPs summarize key nutritional aspects and characteristics of the foods [25]. For example, FOPs may provide details on a few nutrients (e.g., calories, fat, and sodium) or an overall nutritional value.

Some research in this area has shown a positive impact of food labels on consumer understanding of product nutrition information and food choice [17,26,27]. For example, in two separate randomized controlled trials, researchers found that individuals were able to use FOPs to select healthier foods [23,24]. On the other hand, a few studies have shown that the availability of FOPs on products had no effect on food choice using the Traffic Light system [28,29]. Similarly, the availability of single-summary FOPs (Smart Choices) had no effect on the amount of cereal consumed in a laboratory study [30]. Thus, it could be that when individuals are directed to answer questions based on FOP information, they are able to do so; but when they are not, the FOPs are not useful. However, it remains unclear from these studies whether the FOP information goes unnoticed or is noticed but is not used to improve dietary choice.

Several studies have examined the association between food label use and food choice using objective measures of food label use, in particular, those that involve eye-tracking methodology [22,31,32,33,34,35]. One study, for example, showed that individuals pay little attention to nutrition tables relative to traffic-light FOP symbols [22]. Another study compared self-reported use of food labels with the attention to food labels in a simulated shopping task [34]. The researchers found that subjective and objective measures of food label use were not well aligned with each other; however, dietary quality was not assessed, making it unclear how well either measure predicted quality of diet. Self-reports of food label use represent perceptions based on memory for specific behaviors, which may or may not be accurate. The nature of these perceptions regarding food label use could be related to motivation to maintain or improve the quality of one’s diet. For example, food label use, based on self-reported measures, is related to attitudes toward healthy eating, such as stage of change associated with dietary practices [36]. This is consistent with other work showing that stages of change are related to dietary changes [37,38].

The present study examined self-reported and objective use of food labels to determine if they had comparable associations with dietary quality. Objective food label use was operationalized as individuals’ attention to nutrition information on food labels during a mock shopping task. Individuals viewed the fronts of two food packages, each containing FOPs that differed in nutrient content, while their eye movements were monitored using eye-tracking methodology. Eye movements have been used in past research to infer decision making processes in general [39] as well as within context of diet and health [40]. We compared both measures of food label use to stage of change. Our goals were to determine (1) whether self-reported label use and objective label use showed similar relationships to dietary quality; (2) the degree to which objective and subjective measures overlapped; and (3) whether stage of change was more closely related to self-reported or objective measures of label use.

## 2. Experimental Section

### 2.1. Sample

We used stratified cluster sampling to recruit 1891 households in the Sacramento area in 2013–2014 with publicly available phone numbers. 1286 individuals were contacted by phone and 238 were excluded due to report of neurodegenerative disease, head trauma, stroke, or lack of fluency in English, or rarely or never buying groceries for their household. 392 individuals agreed to participate in the study and met the study criteria. Due to poor quality eye-tracker calibration for 34 individuals, the final sample included 358 participants, ages 20–78, mean age = 49.9. The Institutional Review Board of the University of California, Davis, approved the study and free and informed consent of participants was obtained.

### 2.2. Materials and Equipment

**Figure 1:**
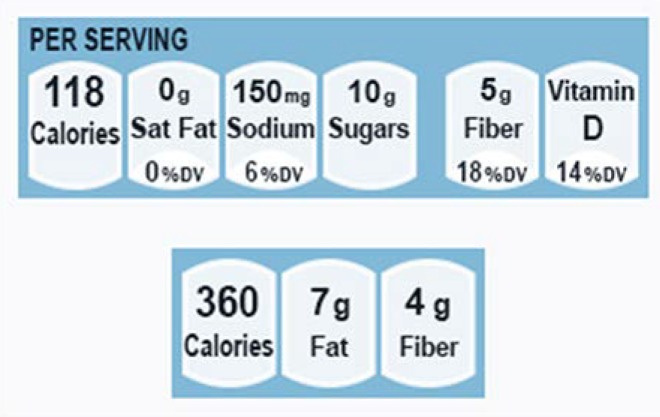
Front of Package (FOP) symbols for cereals (**top**) and frozen entrées (**bottom**).

The mock shopping task consisted of 24 pairs of similar cereal or frozen entrée products. These product types were selected because these foods often have Front of package symbols (FOPs) on their package fronts, most commonly six cells for cereals and three cells for frozen dinners [33]. The FOPs on cereals contained percent daily values (%DV) and/or amounts (grams or milligrams) per serving of saturated fat, sodium, sugar, fiber, and vitamin D, as well as calories per serving. FOPs for frozen dinners contained cells for calories, fat, and fiber. For cereals, we used the Facts Up Front format style promoted by the Grocery Manufacturers Association and the Food Marketing Institute, as this format currently appears on most cereal products in the US. For frozen entrees, we used a format that represents a blend of those appearing on frozen entrée packages (e.g., Smart Ones, Marie Callender). Samples of the types used in the present study are shown in Figure 1.

Eye movements were assessed using an EyeLink 1000, which has a sampling rate of 1000 Hz and average accuracy of 0.25–0.5 degree. The eye tracker projects an infrared light onto the participant’s eye, which reflects back to a video camera that records time and eye-position coordinates. The task began with a nine-point calibration task followed by two practice comparisons.

### 2.3. Measures

#### 2.3.1. Stage of Change

A 3-item measure of stage of change was based on the Transtheoretical Model of Change [41]. The items included individuals’ readiness to reduce saturated fat consumption, reduce junk food consumption, and increase fruit and vegetable consumption [40,42]. Participants were asked to select the response that “best represents your perspective/behavior on eating ___” on a scale of 1 (never think about it) to 6 (have been doing this longer than 6 months). Scores were averaged across the three items.

#### 2.3.2. Self-Reported Food Label Use

Items (*n* = 13) on food label use were taken from the Food and Drug Administration’s 2008 Health and Diet Survey [43]. The survey included items such as “People tell us they use food labels in many different ways. When you consider eating a product for the first time, either in the store or at home, how often, if at all, do you use the labels in the following ways?” for 8 goals including the comparison of different foods. The measure showed good internal consistency, alpha = 0.91. Responses were scored on a 5-point scale (5 = Always, Often, Sometimes, Rarely, 1 = Never) and averaged.

#### 2.3.3. Dietary Quality

Nutrient intake was estimated using the Automated Self-Administered 24-h Dietary Recall (ASA24) system, version 2011 and 2014, developed by the National Cancer Institute. This system prompts individuals to recall, in detail, for each meal and snack, the foods and beverages they have consumed during the previous 24 hour period. The program calculates kcal and the macronutrients (e.g., fats, protein, carbohydrates) as well as 90 micronutrients (vitamins, minerals), food components (e.g., grains, vegetables), and others (e.g., added sugars). The Healthy Eating Index (HEI) was calculated, as a measure of overall dietary quality with a maximum score of 100.

### 2.4. Procedure

For the mock shopping task, high-resolution images of package fronts were presented side by side on a wide screen monitor. Participants were asked to select their preferences by using a mouse to click on the product they wanted to purchase. Participants first completed a general demographic questionnaire, which included health, height, and weight items, followed by a vision assessment (to verify their self-reported vision asked during the screening process), the stage of change measure, brand sensitivity and brand familiarity (not included in this study), and the food label use survey Participants completed the dietary quality assessment at the end of the session and then again roughly two weeks later.

### 2.5. Data Analysis

Objective food label use was operationalized as proportion dwell time in each FOP nutrient interest area (3 for frozen entrees, 6 for cereals), reflecting attention to nutrition information on the food labels. Proportion dwell time represents the viewing time for each interest area as a proportion of total time for that food choice. For each subject, we calculated the average proportion dwell time for each nutrient and then summed across the averages. We entered the sums into linear regression models to examine the relationship between attention to nutrition information on food labels and self-reported label use on the one hand, and dietary quality on the other. This relationship was examined after controlling for stage of change, as well as demographic variables of age, sex, body mass index (BMI), income, and education, first by fitting a model with just the demographic variables, and then adding the attention, self-reported label use, and stage of change variables. Model assumptions were validated using graphical examination of the residuals. Potential problems with multicollinearity were examined by estimating all pair-wise correlations for the covariates in the model. Finally, the predicted sum of squares statistic was used to test for the best model.

## 3. Results

A histogram of the residuals was symmetric and approximately bell-shaped and a scatter plot of the residual by predicted value showed no trends or patterns, providing no indication of problems with the normal or linearity assumptions. No pair of covariates had a correlation with an absolute value higher than 0.5, providing no indication of problems with multicollinearity. Approximately 60% of participants were women, with an average age of 50 years and an average of 16 years of formal education (Table 1). About 37% of participants were overweight (24.9 < BMI ≤ 29.9), and 29% were obese (BMI > 29.9). About 74% of participants were white. The mean HEI in the sample was 56 (95% CI 54.6, 57.0), slightly better than national averages based on NHANES (50 for men, 53 for women) [44], but with substantial variation (range 28–90 of maximum 100). Participants also reported a wide range of stage of change dietary habits, but with an average of 4.6, suggesting widespread interest in selecting healthier foods. Self-reported food label use was also substantial (mean 3.6, 95% CI 3.52, 3.68) but not universal (range 1 to 4.8 on scale of 1 to 5, where 1 = never and 5 = always).

**Figure 2:**
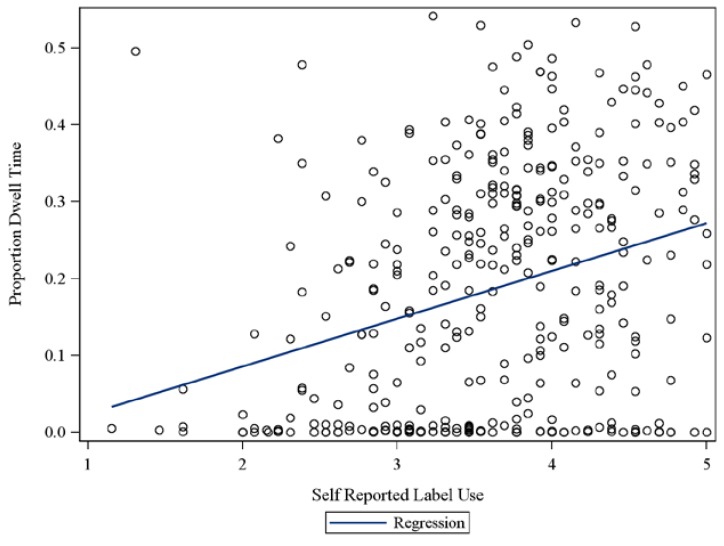
Objective Food Label Use (proportion dwell time) by Self-Reported Food Label Use.

The average proportion of dwell time devoted to FOPs (Table 1), the objective measure of label use, was 0.186 (95% CI 0.17, 0.20) indicating that, on average, individuals spent 18.6% of their total viewing time for that food choice on nutrition information on package fronts (FOPs). However, there was considerable variation, ranging from 0% to 54% of total time per choice. The two measures of food label use were moderately related (*r* = 0.29, *p* < 0.001), but there was considerable variation as shown in Figure 2. Thus, the two measures of food label use were not assessing identical behaviors.

**Table 1:** Demographics and Summary Statistics.

Variable	*N*	Mean (SD) or Percent (%)
*Age*	392	49.9 (16.5)
*Education (years)*	392	15.8 (2.5)
*Sex (Female)*	392	60.0%
*Single*	390	46.9%
*Weight Status*	373	
Overweight (24.9 < BMI ≤ 29.9)		36.7%
Obese (BMI > 29.9)		29.2%
*Parental Status (had children)*	390	71.5%
*Race*	390	
White		74.0%
African American		9.7%
Other		16.3%
*Income*	389	
Less than $10,000		4.9%
$10,000 to $14,999		6.4%
$15,000 to $24,999		6.2%
$25,000 to $34,999		8.7%
$35,000 to $49,999		14.4%
$50,000 to $74,999		19.3%
$75,000 to $99,999		18.0%
$100,000 to $149,999		15.2%
$150,000 to $199,999		4.6%
$200,000 or more		2.3%
*Self-Reported Food Label Use*	392	3.8 (0.8)
*Objective Food Label Use—Proportion Dwell Time*	358	0.186 (0.16)
*Stage of Change*	390	4.57 (1.1)
*Healthy Eating Index (HEI)*	392	55.8 (12.5)

Dietary quality as assessed by the Healthy Eating Index (HEI) was positively associated with both self-reported and objective measures of label use in univariate models. Sex, BMI, and education were all significantly associated with HEI in both univariate and multivariate models. Income was not significant after controlling for age, sex, and education (*p*-value = 0.78), so was omitted from further models. After adjusting for age, sex, BMI, and education, self-reported and objective measures of label use were independently and significantly associated with HEI (Table 2). Average HEI increased 0.138 points for every 1 percentage point increase in proportion FOP dwell time (*p* < 0.001), and 2.9 points for every 1 point higher on the self-reported label use scale (*p* < 0.001). Dietary quality increased with age over the range studied (1.4 points for every 10 years older, *p* < 0.001). As in national studies, women reported better dietary quality (2.6 points better, *p* = 0.031), and higher education also predicted better diet (0.76 points for every additional year of education, *p* = 0.003). Those with higher BMI tended to report worse dietary quality (0.47 points lower for every one-point increase in BMI, *p* < 0.001), regardless of label use, education, sex, and age.

**Table 2:** Covariates of the Healthy Eating Index, Linear Regression Results.

Parameter	Estimate	SE	t Value	Pr > |t|
Intercept	38.3	5.47	7.01	<0.001
Age (years >20)	0.143	0.037	3.88	<0.001
BMI	−0.467	0.094	−4.97	<0.001
Education (years)	0.759	0.252	3.01	0.003
Sex: Female (Male = ref)	2.62	1.21	2.16	0.031
Self-Reported Food Label Use	2.88	0.807	3.57	<0.001
Objective Food Label Use (Proportion Dwell Time)	13.8	3.9	3.50	<0.001

We next examined associations between motivation, as assessed by the stage of change in eating healthy foods, and both measures of food label use. Stage of change was moderately associated with self-reported label use (*r* = 0.48, *p* < 0.001) as well as with objective attention paid to FOPs (*r* = 0.37, *p* < 0.001). This finding suggests that people may be operationalizing their nutrition goals in part by reading labels. Table 3 shows that greater stage of change (commitment to following or improving diet) was associated with better current dietary quality, with a 2.4-point increase in quality for every 1-point higher commitment (*p* < 0.001). Objective measurement of attention to nutrition information predicted HEI independently of stage of change, with little change in its impact on dietary quality. However, the association with self-reported label use was attenuated, with about half its previous association accounted for by stage of change, and the remaining association (with HEI) no longer statistically significant. As before, dietary quality was higher for women than men, improved with age and education, and was lower for those with higher BMI.

**Table 3:** Covariates of the Healthy Eating Index, Linear Regression Results Adding Stage of Change.

Parameter	Estimate	SE	T Value	Pr > |t|
Intercept	33.2	5.54	6.00	<0.001
Age (years >20)	0.124	0.037	3.38	<0.001
BMI	–0.421	0.093	–4.52	<0.001
Education (years)	0.687	0.249	2.76	0.006
Sex: Female (male = ref)	2.69	1.19	2.26	0.025
Self-Reported Food Label Use	1.50	0.877	1.71	0.089
Objective Food Label Use (Proportion Dwell Time)	10.7	3.95	2.71	0.007
Stage of Change	2.41	0.651	3.70	<0.001

## 4. Discussion

Establishing a connection between food label use and dietary intake is important [45]; however, studies that have included objective measures of food label use are particularly rare. Data from the present study adds to past work showing that food label use is related to dietary quality [17,46] and choice [47] by showing that objective measures of use are also strongly tied to the quality of dietary intake. Individuals who paid more attention to nutrition information on package fronts were more likely to consume a healthy diet. It is important to note that it is not possible to determine whether there is a causal effect using this type of experimental design and biases (e.g., social desirability) are possible [48]. Still, the findings lend support to the idea that food label information is being utilized to consume healthier foods.

In general, these findings are consistent with research based on self-reported use of food labels. Several studies have shown that more frequent label users eat a healthier diet than do less frequent label users [10,15]. We found that 6.6% of variance in dietary intake was explained by self-reported food label use which is consistent with past work indicating that 2% to 17% of the variance is explained [12]. Our data add to the literature by showing that objective measures of food label use (shown here as attention measured while purchasing decisions were being made) also show a connection to dietary quality. Our results showed that 8.5% of variance in dietary intake was explained by attention to nutrition information on food labels. Although the two approaches to food label use are related to each other and have a comparable degree of association to dietary quality, they are not identical. This was evident in their moderate bivariate association (*r* = 0.29).

The findings also add to the literature by suggesting that self-reported food label use has a stronger motivational component than does attention to food labels in a mock shopping task. We found that the regression coefficient for self-reported food label use became marginal when stage of change was added to the model, however, the coefficient for the objective measure remained unchanged. This suggests that, when individuals think of the frequency with which they use food labels, they may also be considering their readiness to change their diet. In this way, the notion of food label use may be associated with a healthful eating belief system that is not necessarily evident in actual behavior. Similarly, the finding could indicate that when individuals report using food labels, they may be incorporating intentions to eat healthier foods in their rating. Both of these possibilities may offer an opportunity for food labels to play a larger role in closing the gap between intention and consumption.

To be sure, not all past work has shown a connection between food labels and dietary choice. Several factors may contribute to the apparent inconsistencies in the literature. One study, for example, found that the presence of FOPs on a cereal box did not influence cereal consumption [30]. The study investigated a highly simplified FOP design, Smart Choice, which does not provide sufficient nutrition-specific information [21]. Participants in that study were also not offered different choices, so did not have an opportunity to compare products. Another important factor to consider is that the earlier study examined consumption in one meal whereas other studies examine a wider range of eating occasions, for example, as assessed by two, 24 hour dietary recalls. It is important to note that participants in the earlier study as well as in the present study were not given any instructions (or suggestions through a prior task) to look at nutrition information. In this way, individuals were free to consider nutrition information if it was important to them, but were not prompted or encouraged to do so.

The current findings show promise for the US government’s intention to promote healthful food choices through food labels. As outlined in the Dietary Guidelines of 2010 (and undoubtedly those to be released in 2015), Americans are urged to reduce intake of certain foods and food ingredients such as sodium, solid fats and added sugars and to increase intake of nutrient dense foods in an effort to decrease diet-related chronic disease risks and prevent current trends in weight gain and obesity development. The findings also show promise for FOPs to communicate important nutrition information. The Institute of Medicine stated that prominent placement and simplified format FOPs should offer consumers more readily noticeable and usable information [25]. The data presented here suggest that the first part of that goal, noticeability, is being met. More specifically, our data provide evidence that FOP information is important to consumers’ dietary decision making, consistent with similar research in fast food restaurants [49], and highlights the need to ensure that only important and relevant information is provided. For example, it may be less important to present sodium on cereal than it is on soup packages, and similarly, less important to present total fat than saturated fat on many products. Although manufacturers must be in compliance with FDA regulations regarding the NFP, and FOP information must reflect the information in the NFP, manufacturers decide which nutrients to highlight on the package front. At present, there are no governmental regulations in the US surrounding how that is done. Policy makers rely on science-based consumer research when writing regulation regarding food labeling. Our data inform policy makers by showing that abbreviated nutrition information that appears on package fronts is important for those who consume more healthful diets and therefore regulations should be designed to ensure that only the most relevant type of information be presented on package fronts.
